# An integrative analysis identifying transcriptional features and key genes involved in COVID-19

**DOI:** 10.2217/epi-2020-0168

**Published:** 2020-11-26

**Authors:** Guoping Li, Junyi Wang, Xiang He, Lei Zhang, Qin Ran, Anying Xiong, Dehong Wu, Lingjuan Hu, Qi Song, Dong Zhu

**Affiliations:** ^1^Laboratory of Allergy & Inflammation, Chengdu Institute of Respiratory Health, The Third People's Hospital of Chengdu, Affiliated Hospital of Southwest Jiaotong University, Chengdu 610031, China; ^2^Department of Pulmonary and Critical Care Medicine, Sub-center of National Clinical Research Center for Respiratory Disease, The Third People's Hospital of Chengdu, Affiliated Hospital of Chongqing Medical University, Chengdu 610031, China; ^3^State Key Laboratory of Quality Research inChinese Medicine, Macau University of Science & Technology, Taipa, Macau(SAR), China; ^4^Department of RespiratoryDisease, Renshou county people's hospital, Chengdu 620550, China; ^5^Department of Respiratory Disease, Sichuan Friendship Hospital, Chengdu 610000, China

**Keywords:** COVID-19, *LCN2*, SARS-CoV-2, *STAT1*, transcriptional features

## Abstract

**Aim:** To elucidate the transcriptional characteristics of COVID-19. **Materials & methods:** We utilized an integrative approach to comprehensively analyze the transcriptional features of both COVID-19 patients and SARS-CoV-2 infected cells. **Results:** Widespread infiltration of immune cells was observed. We identified 233 genes that were codifferentially expressed in both bronchoalveolar lavage fluid and lung samples of COVID-19 patients. Functional analysis suggested upregulated genes were related to immune response such as neutrophil activation and antivirus response, while downregulated genes were associated with cell adhesion. Finally, we identified *LCN2*, *STAT1* and *UBE2L6* as core genes during SARS-CoV-2 infection. **Conclusion:** The identification of core genes involved in COVID-19 can provide us with more insights into the molecular features of COVID-19.

Coronavirus disease 2019 (COVID-19) caused by a novel severe acute respiratory syndrome coronavirus-2 (SARS-CoV-2) is a global pandemic. To date, there are more than 2 million confirmed cases globally, and the number is still growing. Most COVID-19 patients exhibit mild or moderate symptoms, such as cough and fever, but around 15% of patients could progress to severe pneumonia, acute respiratory distress syndrome (ARDS) or multiple organ failure [1,2]. Host immune responses play a critically important role in the battle against the virus. However, dysregulation of the release of inflammatory cytokines, known as cytokine storm, correlates with disease severity and prognosis of patients [3,4].

There are no effective antiviral treatments or vaccines currently available against SARS-CoV-2. Elucidating the pathogenesis of SARS-CoV-2, especially the transcriptional alterations in host post infection is indispensable for winning the battle. Recently, Xiong *et al.* utilized the RNA-Seq approach to depict the transcriptional features of bronchoalveolar lavage fluid (BALF) and peripheral blood mononuclear cell (PBMC) samples of COVID-19 patients and found key cytokines such as CCL2/MCP-1, CXCL10/IP-10 and CCL3/MIP-1A were excessively released in COVID-19 [5]. Moreover, Blanco-Melo *et al.* compared the characteristics of host transcriptional response of SARS-CoV-2 with other viruses and identified unique features of SARS-CoV-2 in *in vitro*, *ex vivo* and *in vivo* systems [6]. These findings provided us with the in-depth knowledge of SARS-CoV-2 infection, but more is still needed.

Joint analysis of different individual studies is gradually becoming a popular and common approach to investigate the key features of diseases [7]. As the outputs of individual experiments may be noisy, it is important to look for findings that are supported by several pieces of evidence to promote the reliability. Here, we integrated the RNA-Seq data from different individual studies of COVID-19 to systematically evaluate the transcriptional characteristics of this disease. We first compared the immune cell infiltration features in BALF, lung and PBMC samples of COVID-19 patients. Then, we identified some codifferentially expressed genes, which were associated with immune response and cell adhesion. Moreover, we found three core genes, *LCN2*, *STAT1* and *UBE2L6*, were consistently upregulated across seven independent studies of SARS-CoV-2. Our findings could provide more insights into the molecular mechanisms of this pandemic.

## Materials & methods

### Data collection & differential expression analysis

RNA-Seq count data of four BALF samples and three PBMC samples from three COVID-19 patients were obtained from a recently published study [5]. RNA-Seq count data of normal human bronchial epithelium (NHBE), transformed lung alveolar (A549) cells, transformed lung alveolar (A549) cells transduced with a vector expressing ACE2, Calu-3 cells and COVID-19 patient samples were downloaded from the Gene Expression Omnibus (GEO) data repository under the accession number GSE147507 (www.ncbi.nlm.nih.gov/geo/query/acc.cgi?acc=GSE147507). Above cells were infected with SARS-CoV-2. Differential expression analysis was conducted in the R computing environment using the DESeq2 R package [8]. Gene expression profiles of smoking individuals and SARS-CoV infected cells were also downloaded from the GEO database (GSE63127, www.ncbi.nlm.nih.gov/geo/query/acc.cgi?acc=GSE63127 and GSE17400, www.ncbi.nlm.nih.gov/geo/query/acc.cgi?acc=GSE17400). 59 RNA-Seq of The Cancer Genome Atlas (TCGA) lung adenocarcinoma paired normal samples were downloaded from Xena datahub (https://xenabrowser.net/, TCGA Pan-Cancer cohort).

### Immune cell abundance quantification

The count data of BALF, lung and PBMC samples were converted to counts per million) followed by TMM normalization using the edgeR R package [9]. The TMM normalized values were used for immune cell abundance quantification. The single sample Gene Set Enrichment analysis (ssGSEA) algorithm was used. The cell markers of 20 immune cell types were manually collected from CellMarker database (http://biocc.hrbmu.edu.cn/CellMarker/).

### Functional annotation

Gene ontology (GO) functional enrichment analysis of the consensus gene signatures identified from BALF and lung samples was performed using clusterProfilerR package [10]. To infer the biological functions of *LCN2*, *STAT1* and *UBE2L6*, we included 288 RNA-Seq data of human lung tissues from the GTEx database (downloaded from Xena datahub, https://xenabrowser.net/, UCSC Toil RNA-seq Recompute TPM). The samples were classified into high and low expression groups based on the median expression value of each gene. Then, differential expression analysis was conducted by comparing high-expression group with low-expression group. The fold changes were used as the input for GSEA analysis using clusterProfiler R package with the following parameters: nPerm = 1000, minGSSize = 10, maxGSSize = 500.

## Results

### Immune cell abundance in BALF, lung & PBMC samples of COVID-19 patients

**Figure 1.**
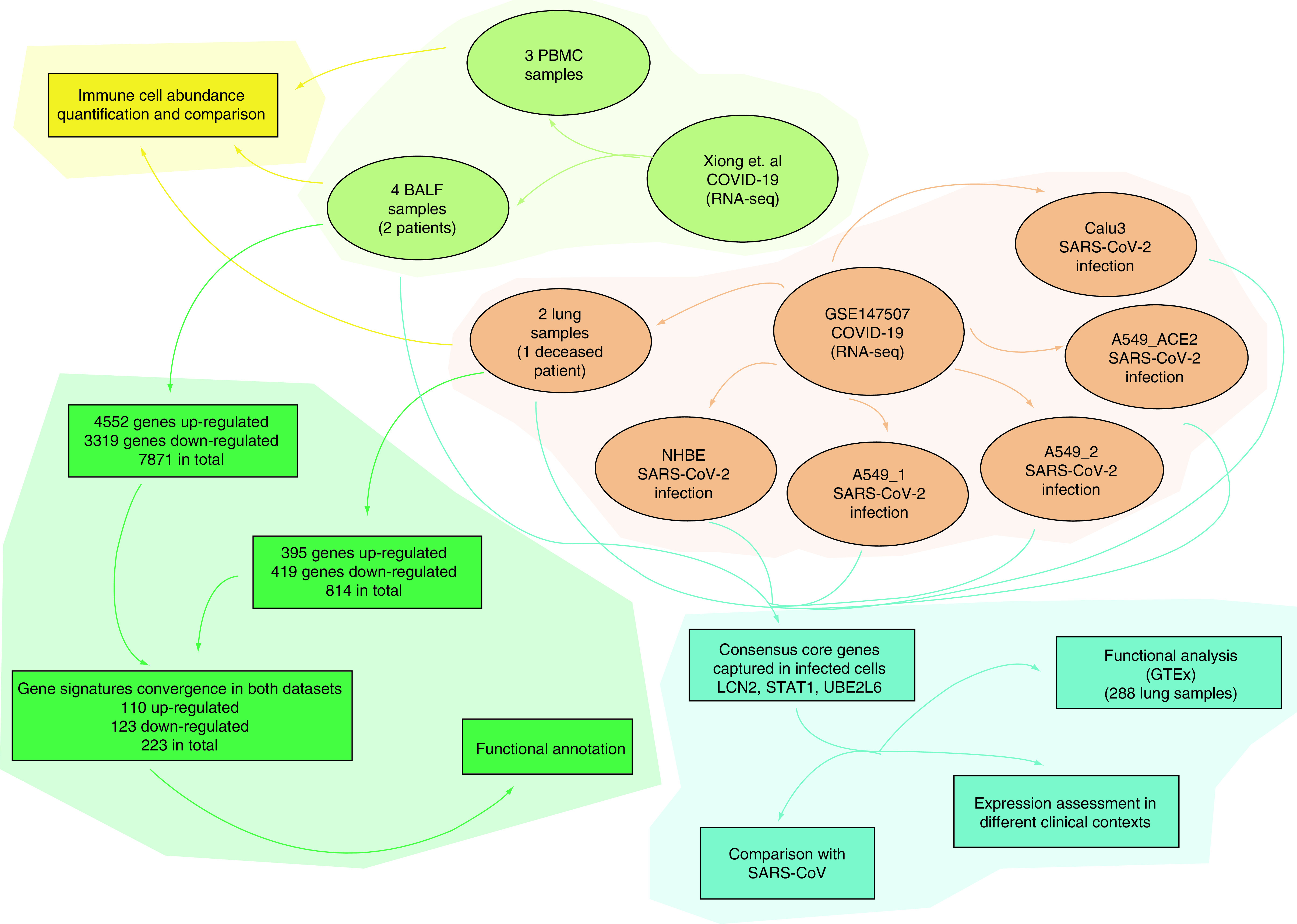
Study flowchart. Two sets of RNA-seq data were analyzed. Xiong's data, in light green, contains two datasets including three PBMS samples and four BALF samples. GSE147507 is in brown, which consists of six datasets, including two lung samples, NHBE SARS-CoV-2 infection, A549_1 SARS-CoV-2 infection, A549_2 SARS-CoV-2 infection, A549_ACE2 SARS-CoV-2 infection, Calu3 SARS-CoV-2 infection. In the yellow part, three datasets (two lung samples, three PBMS samples and four BALF samples) were applied for immune cell abundance analysis. Two datasets, four BALF samples and two lung samples underwent differential expression analysis and function annotation, in green. In the blue panel, seven datasets including four BALF samples from Xiong's data and the six datasets from GSE147507 were used to identify the core genes. BALF: Bronchoalveolar lavage fluid; NHBE: Normal human bronchial epithelium; PBMC: Peripheral blood mononuclear cells.

The study flowchart is illustrated in Figure 1. The dataset from Xiong *et al.* contains the transcriptome information in BALF and PBMC samples of healthy donors and COVID-19 patients (three BALF controls, four BALF samples and three PBMC controls, three PBMC samples) [5]. Another dataset contains the transcriptome information of infected cells, a deceased patient (age 74, two lung samples) and two uninfected human lung biopsies [6].

Both innate and adaptive immune responses can be triggered upon SARS-CoV-2 infection. Understanding the abundance of different immune cell types in the infection sites can help us gain more insights into the immune regulation features in COVID-19. Here, we performed the ssGESA to assess the immune cell abundance in BALF, lung and PMBC samples (Supplementary Table 1). Although the abundance of different immune cell types in different samples of different patients exhibited heterogeneity, some common features existed.

**Figure 2.**
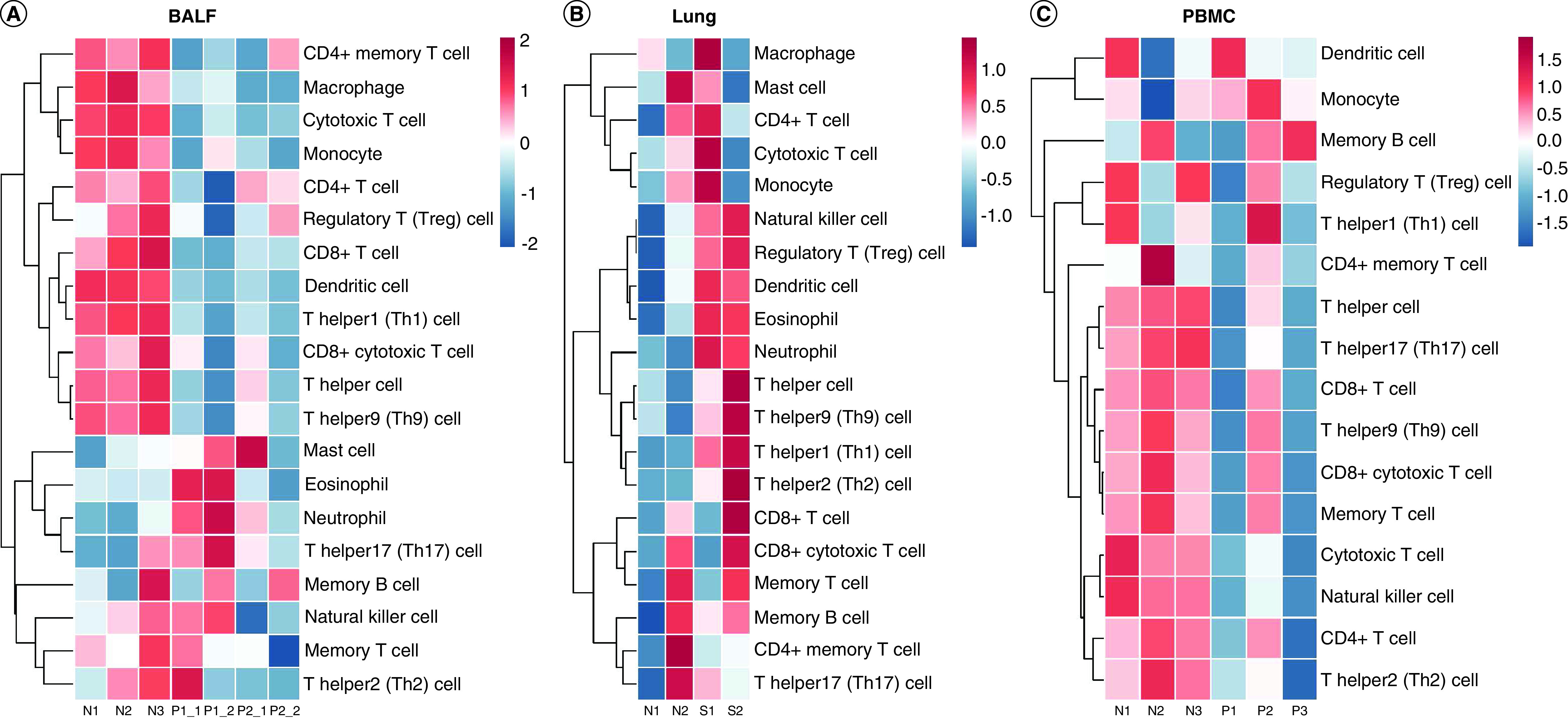
Immune cell types abundance quantification. **(A)** In BALF samples. **(B)** In lung samples. **(C)** In PBMC samples. Blue = Lower abundance; Red = Higher abundance. BALF: Bronchoalveolar lavage fluid; N: Normal control; P: Patient; PBMC: Peripheral blood mononuclear cells; S: Sample.

In BALF samples, decreased levels of macrophages, cytotoxic T cells, monocytes, CD8^+^ T cells, dendritic cells and T helper1 (Th1) cells were found in all four samples of COVID-19 patients compared with BALF healthy control samples. Compared with patient 2, the two BALF samples of patient 1 showed higher abundance of mast cells, eosinophils, neutrophils, Th17 cells and natural killer cells (Figure 2A). Patient 1 is a 74-year-old male, and patient 2 is a 37-year-old male. According to the laboratory reports, patient 1 displayed higher disease severity, the number of lymphocytes in patient 1 was much lower than that of patient 2, of 0.14 x 10^9^/l and 0.64 x 10^9^/l, respectively [5]. Besides, patient 1 had much higher levels of IL-6 and IL-10. Therefore, higher infiltration of immune cells such as neutrophils and Th17 cells may account for the disease severity in patient 1.

In lung samples, higher infiltrations of natural killer cells, eosinophils and neutrophils were also observed in COVID-19. Besides, two samples of this deceased patient displayed consistently higher levels of regulatory T (Treg) cells, dendritic cells, T helper cells, Th9 cells, Th1 cells and Th2 cells. However, some differences also existed in these two samples. For example, the infiltrations of macrophages, mast cells, CD4^+^ T cells, cytotoxic T cells and monocytes were higher in sample 1, while CD8^+^ T cells, CD8^+^ cytotoxic T cells, memory T cells and memory B cells were higher in sample 2 (Figure 2B).

Moving to immune cell abundance in PBMC. The levels of Th17 cells, cytotoxic T cells, natural killer cells and Th2 cells were decreased in all three COVID-19 patients compared with healthy donors, but the decreased level of these immune cells in patient 2 was not that dramatic. Moreover, patient 1 and patient 3 showed a drastic global decrease of lymphocytes such as CD4^+^ T cells and CD8^+^ T cells compared with healthy donors and patient 2 (Figure 2C).

### Convergence of differentially expressed genes from different COVID-19 patients identifying consensus gene signatures

Integrating multiple independent datasets to investigate the complex molecular mechanisms is becoming a preferred method to study the common features of diseases [7]. Here, we compared the differentially expressed genes in BALF and lung samples of COVID-19 patients to systematically evaluate the common transcriptomic alterations in COVID-19.

**Figure 3.**
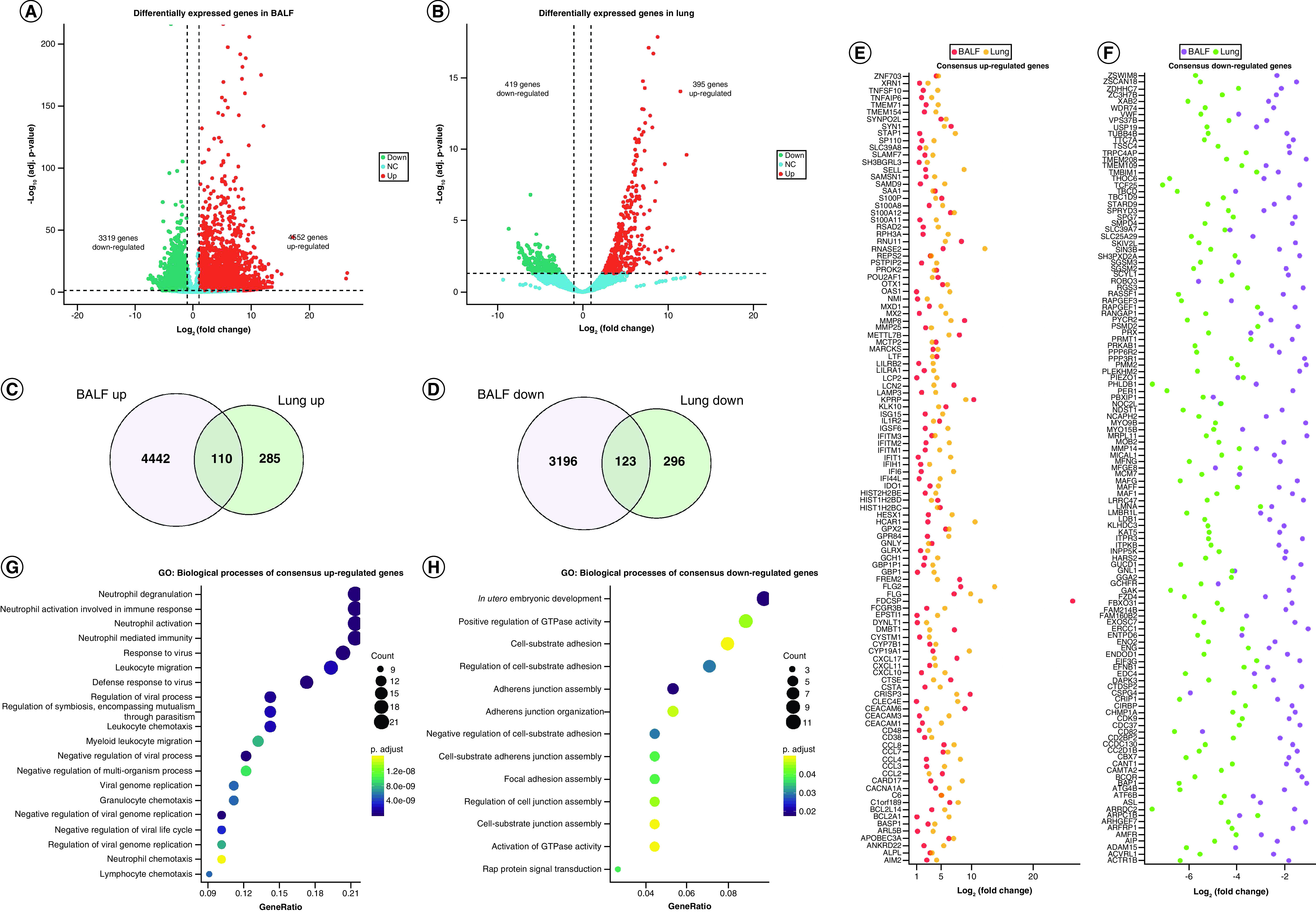
Convergence of differentially expressed genes from different studies of COVID-19. **(A)** Volcano plots showing the number of differentially expressed genes identified from BALF samples. **(B)** Volcano plots showing the number of differentially expressed genes identified from lung samples. **(C)** Intersection of upregulated genes. **(D)** Intersection of downregulated genes. **(E)** The expression alterations of co-upregulated genes in BALF and lung. **(F)** The expression alterations of co-downregulated genes in BALF and lung. **(G)** GO biological process enrichment analysis of co-upregulated genes. **(H)** GO biological process enrichment analysis of co-downregulated genes. BALF: Bronchoalveolar lavage fluid; GO: Gene ontology.

We first performed the differential expression analysis to determine the aberrantly expressed genes in COVID-19 patients (|Log2 fold change| >1, adjusted p-value < 0.05) (Supplementary Table 2). This analysis identified a total of 7871 differentially expressed genes in BALF samples, with 4552 genes upregulated and 3319 genes downregulated (Figure 3A), and a total of 814 genes in lung samples, with 395 genes upregulated and 419 genes downregulated (Figure 3B). Next, we integrated the differentially expressed genes in both datasets to look for intersections. A total of 233 genes were found to be codifferentially expressed in both samples (110 upregulated and 123 downregulated) (Figure 3C & D). The expression changes of the above genes are shown in Figure 3E and F. For example, some key chemokines such as CXCL10/11/17 and CCL2/3/4/7/8, as well as interferon associated molecules including IFITM2, IFITM1, IFIT1, IFIH1, IFI6 and IFI44L were increased in both BALF and lung samples. Functional enrichment analysis of the upregulated genes suggested these genes were mostly related to neutrophil activation (e.g., neutrophil mediated immunity, neutrophil activation and neutrophil degranulation), chemotaxis (e.g., leukocyte migration, leukocyte chemotaxis and leukocyte migration) and antivirus response (e.g., response to virus, defense response to virus and negative regulation of viral process) (Figure 3G, Supplementary Figure 1A & Supplementary Table 3).

On the other hand, functional enrichment analysis of the downregulated genes showed these genes were predominantly enriched in cell adhesion activities such as cell–substrate adhesion, adherens junction organization, focal adhesion assembly, adherens junction assembly and regulation of cell junction assembly (Figure 3H, Supplementary Figure 1B & Supplementary Table 3). On top of that, while most upregulated genes shared the similar fold changes in both BALF and lung samples, the downregulated genes in lung samples of the deceased patient showed a higher level of decrease (Figure 3F). Therefore, we speculated that the degree of the expression changes of these genes may be associated with disease severity.

### Identification of core genes in SARS-CoV-2 infected cells

Elucidating the transcriptional features in the infected cells is critically important for us to understand the pathogenesis of COVID-19. Apart from the two aforementioned bulk RNA-Seq data from COVID-19 patients, we here integrated another five independent SARS-CoV-2 infected cell cohorts to help us dive deeper into the SARS-CoV-2 infected cells. The five independent cohorts consisted of three cohorts of A549 cells (termed as A549_1, A549_2 and A459_ACE2), one cohort of NHBE cells, as well as one cohort of Calu3 cells. The above cells were mock treated or infected by SARS-CoV-2 (three mock-treated and three with SARS-CoV-2) (Supplementary Table 4).

**Figure 4.**
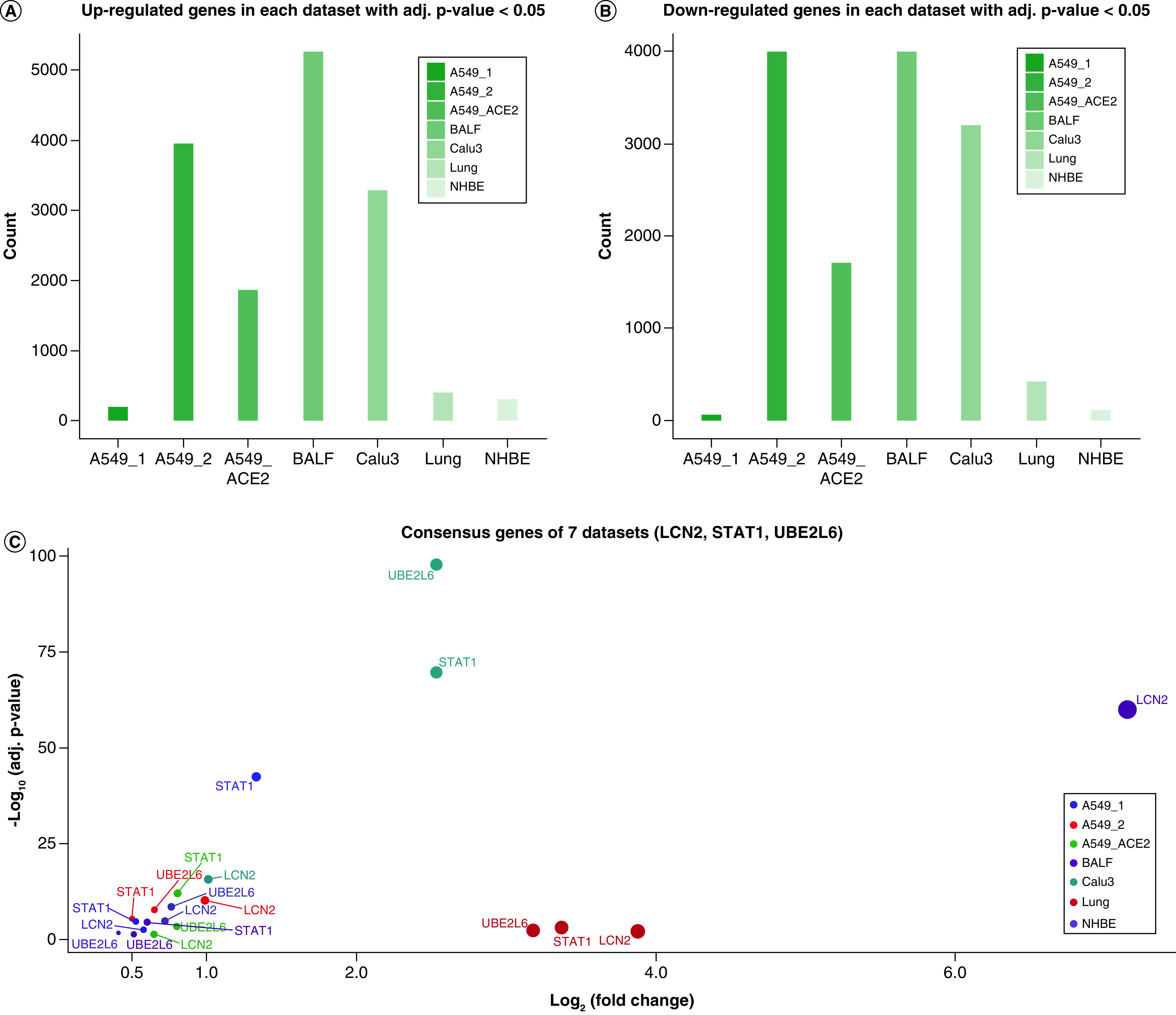
Identification of core genes in SARS-CoV-2 infected cells. **(A)** The number of upregulated genes in each study (adjusted p-value < 0.05). **(B)** The number of downregulated genes in each study (adjusted p-value < 0.05). **(C)** The fold changes of *LCN2*, *STAT1* and *UBE2L6* in each study. BALF: Bronchoalveolar lavage fluid; NHBE: Normal human lung epithelium.

First of all, we analyzed the differential expression of genes in each of the five studies and compared them with bulk RNA-Seq data. We here considered genes with adjusted p-value < 0.05 significant and ignored the fold changes because we wanted to identify more genes that actually played important roles in COVID-19 but were filtered out due to strict criteria. Surprisingly, the number of differentially expressed genes in each individual cell cohort differed significantly (Figure 4A & B). For example, both up- and downregulated genes in A549_1 and NHBE were far less than those of A549_2, A549_ACE2 or BALF (Figure 4A & B). Although the transcriptional features tended to be unique in different datasets, some core genes that displayed the same direction of alteration should exist post infection. Therefore, we integrated above seven studies to search for intersections and we identified three important genes, *LCN2*, *STAT1* and *UBE2L6* (all upregulated). The fold changes of these three genes in each dataset are shown in Figure 4C.

**Figure 5.**
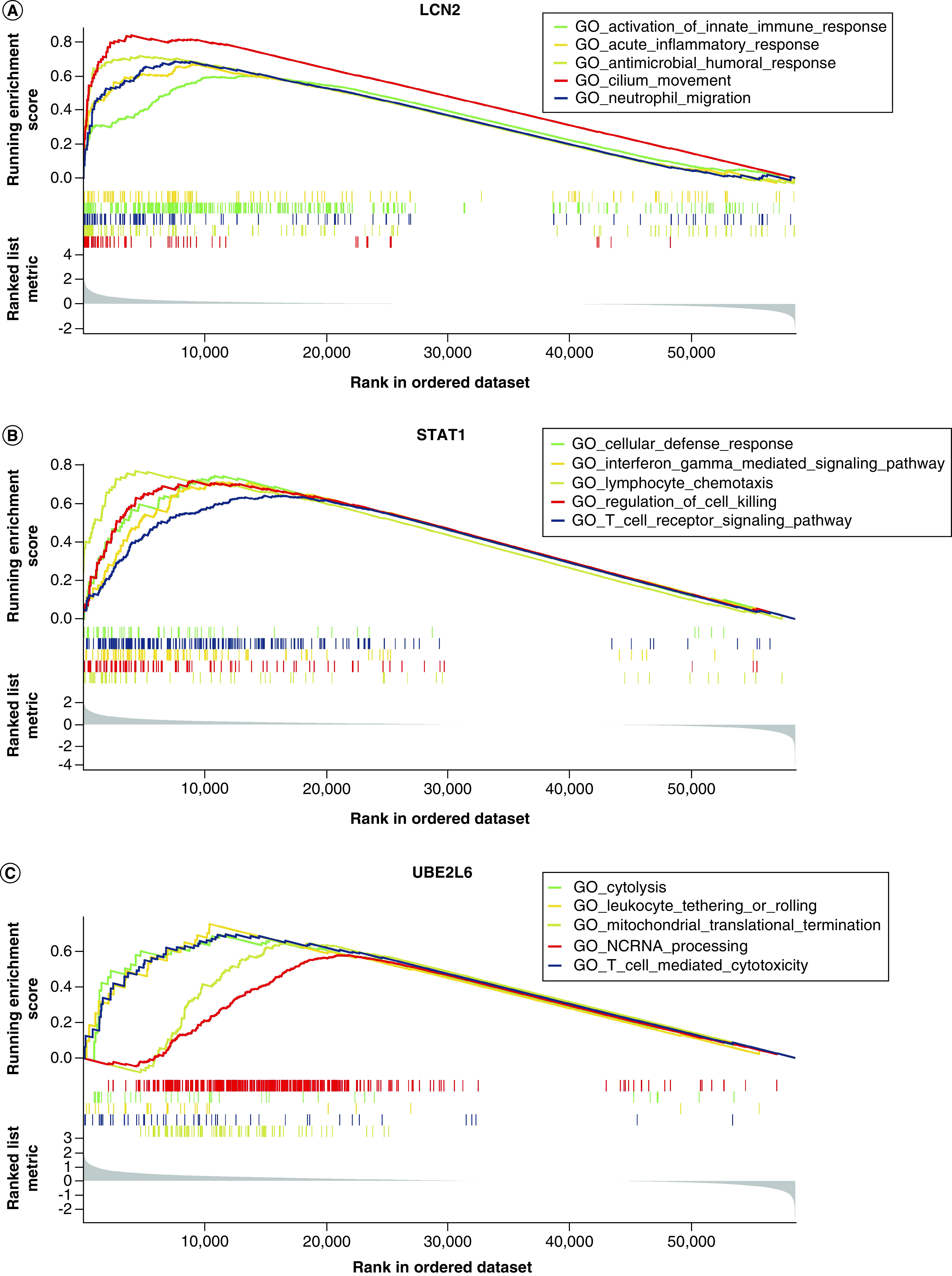
Gene Set Enrichment Analysis of core genes identified. Gene Set Enrichment Analysis showing the biological functions of **(A)** *LCN2*, **(B)** *STAT1* and **(C)** *UBE2L6*. (adjusted p-value < 0.05). GO: Gene ontology.

To investigate the biological relevance of *LCN2*, *STAT1* and *UBE2L6*, GSEA was performed (Supplementary Table 5). Higher expression of *LCN2* was found to be associated with activation of innate immune response, acute inflammatory response, humoral response, cilium movement and neutrophil migration (Figure 5A). For *STAT1*, higher expression groups displayed activities such as cellular immune response, interferon gamma pathway, lymphocyte chemotaxis, cell killing regulation and T cell receptor pathway (Figure 5B). As for *UBE2L6*, we found that it was associated with cytolysis, leukocyte tethering, mitochondrial translational termination, noncoding RNA processing and T cell mediated cytotoxicity (Figure 5C). All the above genes were related to immune responses and some key biological processes such as cytolysis and mitochondrial translational termination, suggesting their important roles in COVID-19.

### Assessing the expression patterns of *LCN2*, *STAT1* & *UBE2L6* in different biological & clinical contexts

Apart from type II alveolar cells, cells in different organs also express ACE2, the entry point of SARS-CoV-2, suggesting the potential risk of infection [11]. Thus, assessing the baseline expression of the core genes in different organs is necessary. Based on the GTEx database, we compared the expression of *LCN2*, *STAT1* and *UBE2L6* across 31 organs and sites in humans. The expression of *LCN2* in lung ranked the third across the human organs and sites (Supplementary Figure 2A). *STAT1* was found to be the highest in lung, and the expression of *UBE2L6* in lung ranked the second compared with other organs and sites (Supplementary Figure 2B & 2C). Moreover, it is noteworthy that the expression variations of *LCN2* were relatively high in organs such as colon and esophagus compared with *STAT1* and *UBE2L6*.

**Figure 6.**
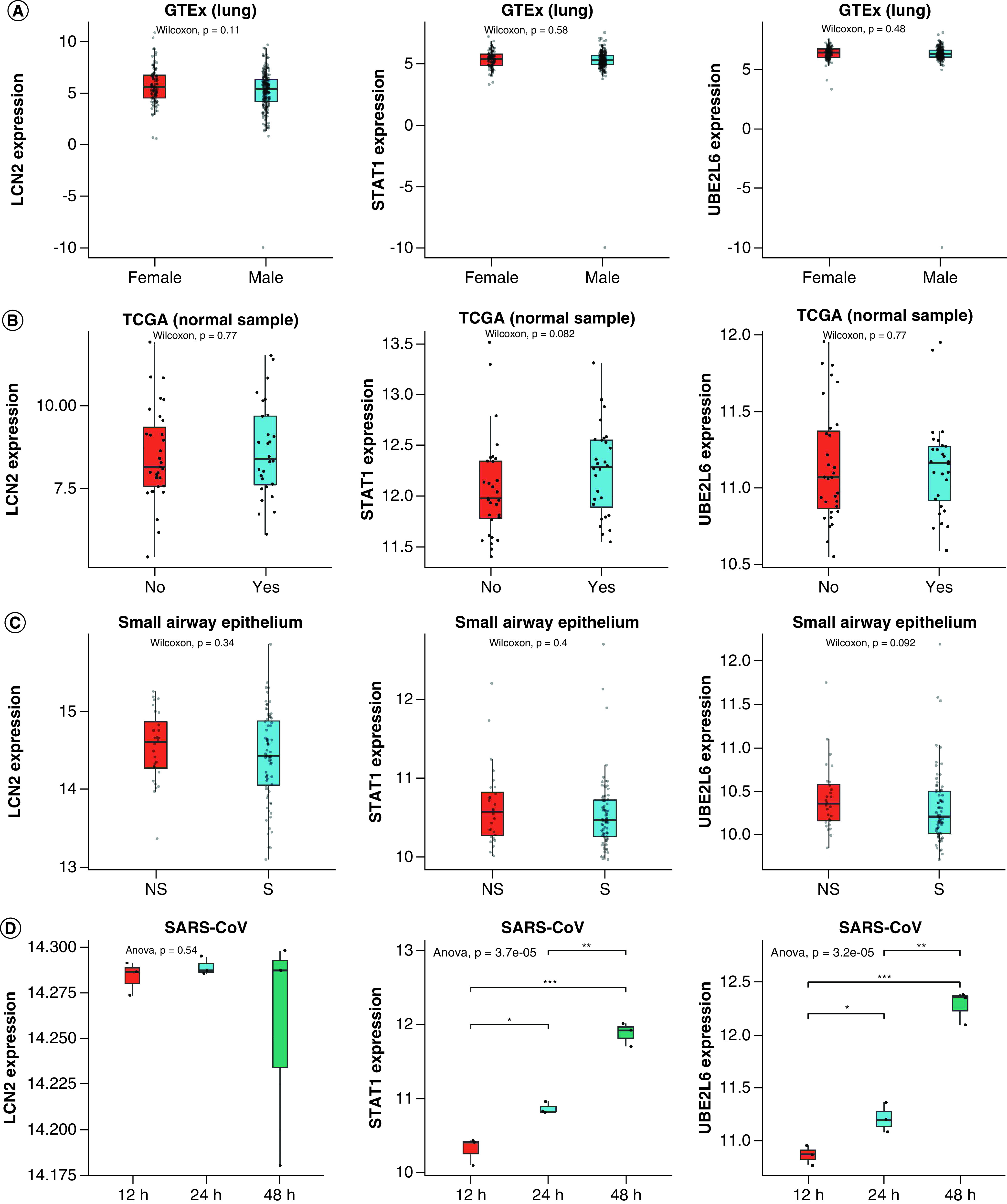
Expression of *LCN2*, *STAT1* and *UBE2L6* in different groups and comparison with SARS-CoV. **(A)** Expression of *LCN2*, *STAT1* and *UBE2L6* in different gender. **(B)** Expression of *LCN2*, *STAT1* and *UBE2L6* taking age into consideration. The median age of this 59-people-cohort is 66. Yes means age greater than 66. No means age less than 66. **(C)** Expression of *LCN2*, *STAT1* and *UBE2L6* in different smoking status. **(D)** Expression of *LCN2*, *STAT1* and *UBE2L6* in SARS-CoV. *p-value < 0.05; **p-value < 0.01; ***p-value < 0.001. TCGA: The Cancer Genome Atlas.

Age is an independent risk factor for COVID-19. Higher expression of ACE2 was found in smoking individuals [12]. Therefore, examining the expression features of *LCN2*, *STAT1* and *UBE2L6* in different clinical contexts such as age, gender and smoking status could help us learn more about the pathogenesis of COVID-19 in different populations. Here, we found that the expressions of *LCN2*, *STAT1* and *UBE2L6* in different gender and smoking status were not significantly different (Figure 6A & C, p-value > 0.05). But a slight higher expression of *STAT1* in the aged population was observed (Figure 6B, middle panel p-value: 0.082).

SARS-CoV and SARS-CoV-2 shared around 80–90% sequence identity [13]. We have identified *LCN2*, *STAT1* and *UBE2L6* as core genes in SARS-CoV-2 infected cells. One question is whether these genes would display sample direction of alterations in both SARS-CoV and SARS-CoV-2 infected cells. We here collected the transcriptome of airway epithelial cells after being infected with SARS-CoV. We found that the expressions of *STAT1* and *UBE2L6* were significantly increased in SARS-CoV infected airway epithelial cells after 24 and 48 h (Figure 6D, middle and right panel, p < 0.05). But the expression of *LCN2* was not significantly altered in SARS-CoV (Figure 6D, left panel, p > 0.05), indicating the possibly unique role of *LCN2* in COVID-19.

## Discussion

COVID-19 caused by SARS-CoV-2 continues to spread across the globe, carrying a mortality of approximately 3.7%. ARDS and cytokine storm syndrome are the leading causes of mortality in patients with COVID-19 [14]. Neutrophilia is regarded as an important risk factor associated with the development of ARDS and progression from ARDS to death [15,16]. Neutrophilia and increased level of neutrophil-to-lymphocyte ratio are reported to be correlated with disease severity and prognosis of COVID-19 patients [17]. Consistently, the BALF sample of patient 1 (higher disease severity compared with patient 2 and patient 3) and the lung sample of the 74-year-old deceased patient displayed a higher level of neutrophil infiltration. Besides, the consensus upregulated genes from BALF and lung samples were also significantly related to neutrophil activities such as neutrophil activation and neutrophil degranulation. Therefore, these data suggested the upregulation of these genes played a key role in neutrophil recruitment in COVID-19. Th17 type responses are found in patients with SARS-CoV-2 and contribute to the cytokine storm. IL-1β and TNF-α, which can promote vascular permeability and leakage [18], are important cytokines involved in Th17 type responses. In the BALF sample of patient 1, we also witnessed a higher level of infiltration of Th17 cells. Taken together, infiltration of immune cells such as neutrophils and Th17 cells could be an important immune phenotype in COVID-19 and collectively contribute to the disease severity in patients with COVID-19.

On the other hand, the downregulated genes were predominantly enriched in cell adhesion activities such as cell–substrate adhesion, adherens junction organization, focal adhesion assembly and adherens junction assembly. Previous studies demonstrated that the downregulated cell adhesion activity was associated with increased permeability of blood vessels. Increased permeability of blood vessels could promote immune cell infiltration, but at the same time, allow more fluid to enter the tissues, resulting in edema [19,20]. In a pathological report of a COVID-19 patient, pulmonary edema was observed [21]. Therefore, downregulation of these genes may also play a key role in the pathophysiology of COVID-19.

The core genes in SARS-CoV-2 infection were identified through integrating five independent *in vitro* studies and two COVID-19 patient studies. We identified three important genes, *LCN2*, *STAT1* and *UBE2L6*. LCN2, also known as neutrophil gelatinase-associated lipocalin, is a mammalian protein expressed by various cell types and is involved in inflammation, ischemia, infection and kidney damage [22,23]. LCN2 can deactivate macrophages and worsen the inflammatory response, resulting in a detrimental outcome of pneumococcal pneumonia [24]. In respiratory syncytial virus infection, overexpression of LCN2 was reported to be associated with more severe viral infection [25]. Here, we found *LCN2* was not only overexpressed in COVID-19 patients but also in infected cells. The GO enrichment analysis network showed that *LCN2* was an important gene that linked neutrophil and virus response activities. Meanwhile, higher expression of *LCN2* was related to inflammatory response, cilium movement and neutrophil migration. Interestingly, expression of *LCN2* was not altered in SARS-CoV infected cells. Therefore, *LCN2* could be an important biomarker in SARS-CoV-2 infection and play a key role in disease progression.

STAT1 is an important regulator in mediating IFN-α/β, IFN-γ and IFN-λ signaling. It was reported that STAT1 could function as a key regulator of wound healing [26]. The defects of STAT1 function may augment viral lung disease by several potential mechanisms [26]. In SARS-CoV infection, the ORF6 of SARS-CoV can block STAT1 translocation into the nucleus, leading to the development of the disease state [27]. In our study, we found that the mRNA level of *STAT1* was increased in SARS-CoV-2 infection. Functional analysis showed that higher expression of *STAT1* displayed increased activities such as cellular immune response, interferon gamma pathway, lymphocyte chemotaxis, cell killing regulation and T cell receptor pathway. Based on the above findings, it can be assumed that the upregulation of the mRNA expression of *STAT1* in SARS-CoV-2 infection could be a protective mechanism. However, a recent study showed that the ORF6 of SARS-CoV-2 shared 85.7% similarity with SARS-CoV [28]. Thus, whether the ORF6 of SARS-CoV-2 could also block STAT1, leading to disease progression, needs further investigation.

**Figure 7.**
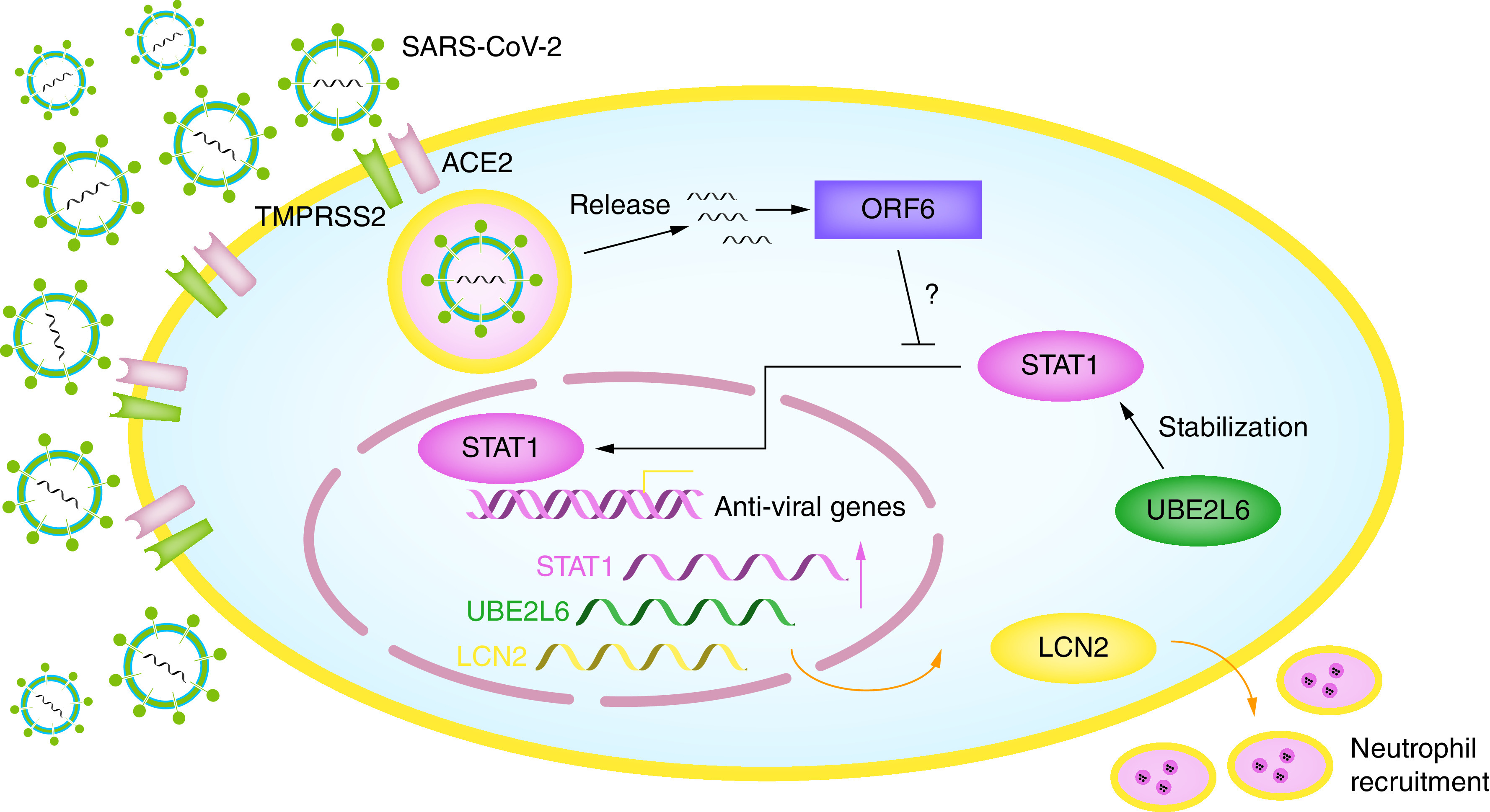
A schematic model of SARS-CoV-2 infection and the role of LCN2, STAT1 and UBE2L6. SARS-CoV-2 virus enters into the host cell, leading to increased gene expression of *LCN2*, *STAT1* and *UBE2L6*. On one hand, LCN2 promotes neutrophil migration and infiltration. On the other hand, STAT1 translocates into the nucleus and facilitates transcription of antiviral genes. Meanwhile, UBE2L6 stabilizes STAT1 to avoid termination of immune response. The ORF6 of SARS-CoV-2 shares about 85% similarity with SARS-CoV, and whether it will block STAT1 remains to be elucidated.

UBE2L6 is a critical enzyme involved in ISGylation [29]. Interestingly, it was reported that ISGylation can stabilize numerous proteins including STAT1 during inflammatory response and inhibit the termination of immune response [30]. As we discussed above, STAT1 is a key regulator in IFN signaling pathway during virus infection. Thus, the possible cross-talk between UBE2L6 and STAT1 in SARS-CoV-2 could be a very interesting aspect (Figure 7).

## Conclusion

In conclusion, our findings identify the phenotype of immune cell abundance associated with neutrophils in COVID-19 patients. Functional enrichment analysis showed that up-regulated genes were enriched in neutrophil activation, chemotaxis and anti-virus response. The cell adhesion activities were lower in down-regulated genes in functional enrichment analysis. LCN2, STAT1 and UBE2L6 were identified as core keys in SARS-CoV-2 infection. Our study revealed the important transcriptional features of COVID-19 and provided us with more insight into the molecular characteristics of COVID-19

## Future perspective

COVID-19, caused by a novel SARS-CoV-2, is a global pandemic. However, there are no effective antiviral treatments or vaccines currently available against SARS-CoV-2. We integrated the RNA-Seq data of COVID-19 and identified *LCN2*, *STAT1* and *UBE2L6* as core keys in SARS-CoV-2 infection. It will be important that the function of the three key genes in SARS-CoV-2 infection should be further investigated. Finally, joint analysis of different individual studies is gradually becoming a popular and common approach to investigate the key features of diseases.

**Summary points**

- We integrated the RNA-Seq data from different individual studies of COVID-19 to systematically evaluate the transcriptional characteristics of this disease.
- We identified 233 genes that were codifferentially expressed in both bronchoalveolar lavage fluid and lung samples of COVID-19 patients.
- Our findings identify the phenotype of immune cell abundance associated with neutrophil in COVID-19 patients.
- Functional enrichment analysis showed that upregulated genes were enriched in neutrophil activation, chemotaxis and antivirus response.
- The cell adhesion activities were lower in downregulated genes in functional enrichment analysis.
- *LCN2*, *STAT1* and *UBE2L6* were identified as core keys in SARS-CoV-2 infection.
- *LCN2* could be an important biomarker in SARS-CoV-2 infection and play a key role in disease progression.
- Our study revealed the important transcriptional features of COVID-19 and provided us more insights into the molecular characteristics of COVID-19.
